# The Coming Control of Syphilis

**Published:** 1913-08-16

**Authors:** 


### THE COMING CONTROL OF SYPHILIS.

It is safe to say that one feature will always stand out to the credit of the Medical Congress now closed, and that is the prominence given to the discussion on the control of syphilis—a discussion held in our largest meeting-place, the Albert Hall, and presided over by Sir Malcolm Morris. A great step has been taken—greater than is realised perhaps at this close view. Public opinion is going to be slowly, yet surely and, above all, permanently, aroused from its lethargic indifference to an evil which is no whit less in its deadliness than others less generally under taboo which are being and have been attacked. True it is that several of the daily newspapers still taboo the word syphilis, but these will soon be forced to give up this feeble evasion.

At the close of the discussion last Saturday two resolutions were put, and the result of the voting showed clearly in which directions changes will have to be made. Compulsory measures will certainly not succeed in lessening appreciably this evil; it is in the direction of providing the means of easily accessible and competent treatment that the first steps must be taken. When such provision is available then is the time to urge the people to submit to be thoroughly cleansed. By gradual education—that is, by the publication, as opposed to the taboo, of important facts—the sense of responsibility and the way in which risks of infection may be avoided will be enforced, and then, but not till then, will it be possible to exert some degree of useful pressure on the ignorant and negligent. Closely akin to the question of notification is that of regulation, and regulation is admittedly a dead failure. Notification, then, is advocated on two grounds—namely, for the purpose of effective treatment and for the purpose of securing instructive statistics. It is mere interference to enact notification unless and until adequate treatment can be promised, and this we are certainly not yet in a position to offer. Even were it not so, any form of compulsion will only lead to concealment, as Continental experience is available to show. On the score of statistical knowledge there is, indeed, something to be said for notification. To ascertain the whereabouts and numerical strength of the enemy are useful tactics. But notification on the lines now in force in dealing with the infectious fevers and tuberculosis is, as applied to syphilis, distinctly premature. For the next ten years only slow progress can be expected in this country with regard to the adequate control of this disease. We believe that nothing more comprehensive than a system of impersonal notification can be successfully carried out. Particulars of cases of syphilis limited to the age and sex of the sufferer, with a few clinical details and family history, would afford a basis on which to estimate the prevalence and local incidence of this infection. Personal notifications to the local authorities of small communities in the nature of things cannot be kept strictly confidential. We throw out a suggestion that all notifications might be sent to some central bureau in the Metropolis. Some overlapping would, of course, be unavoidable, but this already is fairly common with the present system of tuberculosis notification. The only way in which the question of professional secrecy can be overcome is by the education of the public to an increased sense of responsibility. Nothing which will undermine the confidence of the patient in the secrecy of his medical adviser can in the long run be justified. Little more than this small organised effort should be attempted till after a full investigation by the Royal Commission.

